# Interesting Trade-Mark Litigation

**Published:** 1881-10-20

**Authors:** 


### *INTERESTING TRADE MARK LITIGATION.*

The N. Y. Evening Mail, Oct. 15th, says:

Messrs. Allen & Hanburys, a drug house of London, have commenced a suit in the United States District Court, against Parke, Davis & Company, a drug corporation whose manufuctory is in Detroit, but which is also located in this city, to restrain them from the use of the name of the drug "Tonga," and from further selling the drug on the ground that they have a trade mark upon the word "Tonga." The case first came up for hearing yesterday in this city before Commissioner Deuel, and it is attracting considerable interest among the drug trade, as it involves a principle which has frequently been passed upon by the courts of this State but apparently has never been definitely and specifically settled by the Supreme Court; that is, whether a party has the right to trademark the proper name of an article and thus exclude others from the manufacture of the same article, and the name having by adoption and use become the name of the article, whether others have the right to manufacture and sell the same article under the same name, the article not having been patented. This will affect many of the patent medicines and preparations for which protection is sought by registering the names as a trade mark. It is understood that when the case was brought, the complainants, as the chemical extract "Tonga" is of no considerable importance, supposed that Parke, Davis & Co. would consent to cease to use the article, and the case would be dropped. Messrs. Parke, Davis & Co., however, regarded the principle involved in the case as of vital importance to the drug trade, and therefore they will not consent to the settlement of the principle adverse to the ground taken by them by any other court than the court of final resort. Mr. Rowland Cox, of this city, appears for Messrs. Allen & Hanburys, and Mr. Frederick H. Betts; Mr. James Brooks Dill, of thls city, and Judge Lothrop, of Detroit, for Messrs. Parke, Davis & Co.

